# Wild *Vitis* Species as Stilbenes Sources: Cane Extracts and Their Antibacterial Activity against *Listeria monocytogenes*

**DOI:** 10.3390/molecules29153518

**Published:** 2024-07-26

**Authors:** Okba Hatem, Anita Steinbach, György Schneider, Franco Röckel, László Kőrösi

**Affiliations:** 1 Doctoral School of Health Sciences, Faculty of Health Sciences, University of Pécs, H-7622 Pécs, Hungary; okba.hatem@aok.pte.hu; 2 Department of Biochemistry and Medical Chemistry, Medical School, University of Pécs, H-7624 Pécs, Hungary; 3 Department of Medical Microbiology and Immunology, Medical School, University of Pécs, Szigeti Street 12, H-7624 Pécs, Hungary; anitani88@gmail.com (A.S.); schneider.gyorgy@pte.hu (G.S.); 4 Julius Kühn Institute (JKI), Institute for Grapevine Breeding Geilweilerhof, 76833 Siebeldingen, Germany; franco.roeckel@julius-kuehn.de; 5 Research Institute for Viticulture and Oenology, University of Pécs, H-7634 Pécs, Hungary

**Keywords:** bioactive compounds, ε-viniferin, grapevine cane, antibacterial agents, stilbene source, antioxidants, preparative liquid chromatography, genetic fingerprinting

## Abstract

Grapevines (*Vitis* spp.) produce several valuable polyphenol-type secondary metabolites including various stilbenoids. Although the potential application of stilbenes may offer alternative solutions to food safety or health challenges, only little information is available on their antibacterial activity against foodborne pathogens. In this work, high-performance liquid chromatography was used to analyze the stilbenoid profile of various wild *Vitis* species, including *V. amurensis*, *V. davidii*, *V. pentagona*, and *V. romanetii*, selected from the gene bank for grapes at the University of Pécs, Hungary. We found that the stilbene profile of cane extracts is strongly genotype-dependent, showing the predominant presence of ε-viniferin with a wide concentration range ≈ 320–3870 µg/g dry weight. A novel yet simple and efficient extraction procedure was developed and applied for the first time on grape canes, resulting in ε-viniferin-rich crude extracts that were tested against *Listeria monocytogenes*, an important foodborne pathogen. After 24 h exposure, *V. pentagona* and *V. amurensis* crude extracts completely eliminated the bacteria at a minimum bactericidal concentration of 42.3 µg/mL and 39.2 µg/mL of ε-viniferin, respectively. On the other hand, *V. romanetii* extract with 7.8 µg/mL of ε-viniferin resulted in 4 log reduction in the viable bacterial cells, while *V. davidii* extract with 1.4 µg/mL of ε-viniferin did not show significant antibacterial activity. These findings indicate that the ε-viniferin content was directly responsible for the antibacterial effect of cane extract. However, pure ε-viniferin (purity > 95%) required a higher concentration (188 µg/mL) to eradicate the bacteria under the same conditions, suggesting the presence of other antibacterial compounds in the cane extracts. Investigating the onset time of the bactericidal action was conducted through a kinetic experiment, and results showed that the reduction in living bacterial number started after 2 h; however, the bactericidal action demanded 24 h of exposure. Our results revealed that the canes of *V. pentagona* and *V. amurensis* species are a crucial bio-source of an important stilbene with antimicrobial activity and health benefits.

## 1. Introduction

Foodborne diseases are a major public health problem, causing high rates of morbidity and mortality, even in well-developed countries, where food systems are recognized to follow high safety measures [1,2]. For instance, data from the Federal government revealed that 1 in 6 Americans fall sick annually due to foodborne illness, resulting in 48 million patients, 128,000 hospitalization cases, and 3000 deaths each year in the USA [3]. One of the most common foodborne pathogens is *Listeria monocytogenes* (*LM*), a bacterium that causes an infectious foodborne condition known as listeriosis. Invasive listeriosis develops when the bacteria penetrate the intestinal tract, leading to severe symptoms or even death, especially in the elderly and in patients with suppressed immune system. It also poses a risk of miscarriage or stillbirth for women who are infected during pregnancy [4,5,6]. The incidence of listeriosis is relatively low, but in severe cases, the mortality rate can be extremely high, up to 20–30% [7]. The outcome of the infection is not only determined by the immune state of the host, but also the virulence or fitness factors of the infectious agent. During pathogenesis, with the help of adhesion factors, *LM* adheres onto the epithelial cells, and with the help of internalins (InlA, InlB) and the pore-forming toxin listeriolysin O (LLO), evokes its invasion. The internalized bacterium cell escapes from the formed vacuole by using two phospholipases, and by that, enters the cytoplasm of the eukaryotic cell [8]. From this stage, *LM* is able to proliferate in the cytoplasm and can infect adjacent epithelial cells by propelling itself through the plasma membrane of neighboring epithelial cells through the induction of a coordinated actin filament polymerization [9]. This direct cell-to-cell spread is an important issue of *LM* infection, as it can partially avoid the humoral immune system, enhancing its own virulence.

The bacterium is mainly spread through food, such as meat and dairy products contaminated by *LM* originating from the intestinal tract of animals [10]. Consumption of fresh vegetables contaminated by infected animals’ manure can also trigger listeriosis [11,12]. Although *LM* is sensitive to high temperatures, it is resistant to acidic, low water potential, cold, and high salinity conditions [13,14,15,16]. Therefore, foods that are not subjected to heating before consumption, particularly ready-to-eat foods, present a high-potential source of listeriosis outbreaks [17].

Plant-derived polyphenols are receiving growing attention as potential antimicrobial agents and food preservatives [18,19,20]. These secondary metabolites offer numerous advantages over synthetic antimicrobials. Such advantages are that the structural diversity of phytochemical compounds is remarkably high, which increases the possibility of finding unique compounds with beneficial features. Moreover, the biosynthesis of natural compounds is eco-friendly and does not require the expensive precursors and the laboratory equipment that are demanded for synthetic compounds. In addition, many plant polyphenols have been a part of our diet since ancient times. Therefore, their application is safer and more widely accepted by consumers. Polyphenols can be produced in considerable quantities by growing fruits and vegetables, including broccoli, soybean, olive, blueberry, citrus fruits, and grapevine [21]. Notably, grapevine (*Vitis* spp.) is one of the most common fruits globally, with about 7.4 million hectares cultivated in 2018 all over the world, according to the International Organisation of Vine and Wine [22]. The cultivation and processing of grapes result in a huge amount of green waste, such as stems and canes, as well as winery wastes, like grape pomace. These by-products are known to be very rich in valuable polyphenols and display a promising source for further use [23,24]. The phenolic profile of grapevine canes is extensive, and generally comprises phenolic acids, flavonoids (flavanols, flavonols, flavanonols, flavanones), and stilbenoids, with flavonols and stilbenoids being the most abundant groups [25,26]. The concentration of total polyphenols in canes is inconsistent and can vary depending on several factors, such as cultivars [27], genotypes [28], pruning time [26], and storage conditions [29]. One stilbenic compound in the grape family (the *Vitaceae*), namely resveratrol, has been acknowledged as an effective molecule after its positive contribution in the French paradox, i.e., the low occurrence of cardiovascular diseases in France, in spite of high saturated fatty acid intake [30]. Stilbenes are characterized by a C6–C2–C6 skeletal structure, found in a specific number of plant families that possess stilbene synthase enzyme, including the *Vitacea* [31]. As phytoalexins, these compounds are produced in different plant organs under biotic and abiotic stresses [32]. The *Vitaceae* family comprises about 900 species in 14 genera [33], of which 5 genera, including *Ampelopsis*, *Cissus*, *Cyphostemma*, *Parthenocissus*, and one of the most economically important *Vitis*, contain significant amounts of stilbenes [31,34]. Within the *Vitis* genus, about 60–70 species are known. Based on the international literature, about 20 species of *Vitis* have been investigated as potential stilbene sources, with most studies related to *Vitis vinifera* [35]. *Vitis* species contain about 100 mono-, di-, and oligomeric stilbenoids [34]. The most common of these are the various resveratrol derivatives, mainly ε-viniferin, a resveratrol dimer preferentially found in grapevine’s cane and hairy root [36,37]. It has been intensively studied and has numerous positive physiological effects, making it a potentially important compound from nutritional and medicinal perspectives [38]. Health-related properties include antiproliferative, antioxidant, cardioprotective, neuroprotective, and antiaging effects, among others [39,40,41,42]. Research on ε-viniferin antimicrobial efficacy is making progress, with few studies showing encouraging results. In fact, Schnee et al. demonstrated antifungal properties of ε-viniferin by inhibiting the growth of one of the main grapevine fungal pathogens, *Botrytis cinera* [42], and stilbenoid compounds were proposed as promising antifungal agents against mycotoxigenic fungi [43]. With regard to pathogenic bacteria, several stilbenoids that were isolated from *V. amurensis*, including ε-viniferin, showed an antibacterial efficacy against two oral pathogens, *Streptococcus mutans* and *Streptococcus sanguis* [44]. Moreover, ε-viniferin inhibited the biofilm formation of *Pseudomonas aeruginosa* and *Escherichia coli* O157:H7 [45], and synthesized ε-viniferin effectively inhibited *Streptococcus pneumoniae* growth and killed bacteria in biofilms [46], supporting the antibacterial efficacy of this compound. The exact antibacterial mechanism of action is not fully understood, yet it was reported that ε-viniferin may cause cell death by affecting the permeability of the cell membrane [38].

Research on stilbenes related to grapes has been primarily focused on *Vitis vinifera* [35,47]. In this work, we aimed to characterize some wild *Vitis* species, namely *V. amurensis*, *V. davidii*, *V. pentagona*, and *V. romanetii*, whose unique phytochemical profiles are less known, and the available information on their stilbenoids is very limited. In our research, we not only outline the stilbene composition of the aforementioned *Vitis* species, but we also introduce a new extraction method of grape canes for obtaining extracts rich in ε-viniferin. Additionally, we describe a process for purifying the crude extracts using solid-phase extraction and isolating ε-viniferin through preparative chromatography. Finally, we compare the antibacterial effects of crude extracts and ε-viniferin against *LM*. This facultative anaerobic Gram-positive bacterium is highly resistant, and is thus capable of multiplying or surviving in various foods, even in adverse environmental conditions. Our research was motivated by the fact that ε-viniferin-rich cane extracts might be potential antibacterial agents, possessing beneficial physiological and nutritional properties that can support the food and pharmaceutical industry. We are not aware of any other study to report the antibacterial activity of ε-viniferin against *LM.*

## 2. Results and Discussion

### 2.1. Genetic Fingerprinting and Ampelographic Assessment of Grapevine Species

**Figure 1:**
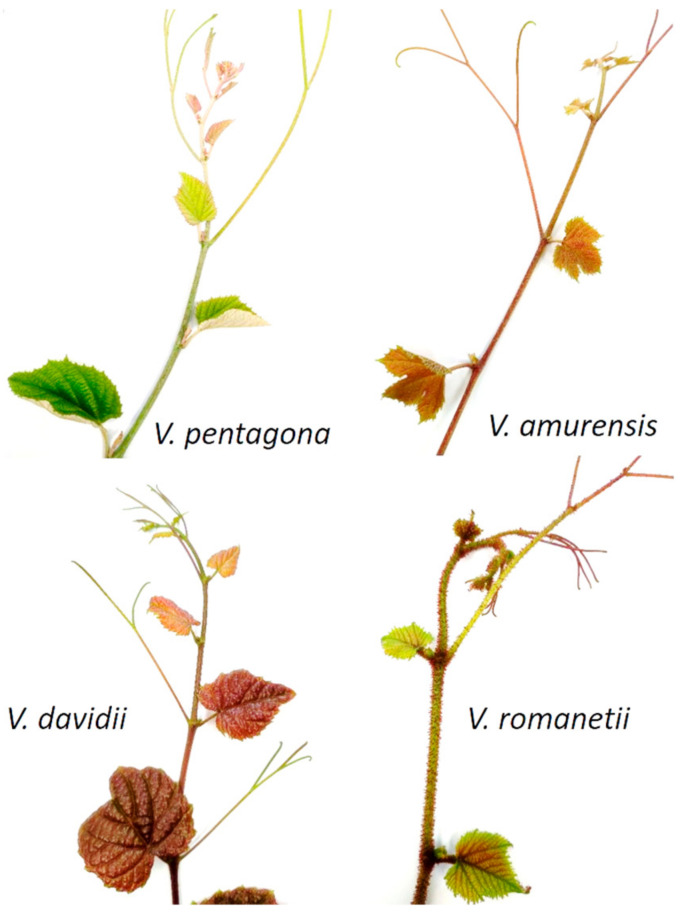
Shoot tips of the studied *Vitis* species.

The studied grape species were selected from the gene bank for grapes at the University of Pécs, focusing on the less-known wild species. Species and/or varietal identity (trueness-to-type) is a prerequisite for ensuring the reproducibility of scientific studies, and therefore is of fundamental importance for breeding and the preservation of biodiversity. In order to ensure the species authenticity of the analyzed individuals, the genetic fingerprint of the nine SSR markers of the *V*IVC (*Vitis* International Variety Catalogue; http://www.vivc.de/ accessed on 15 January 2024) was therefore determined, and an ampelographic comparison was carried out. All four tested individuals showed unique genetic profiles (see Table 1; data compared with the *V*IVC database) with a species-typical morphology, and were consequently confirmed as true-to-type. All four species carry marked ampelographic differences, as shown in Figure 1.

### 2.2. Extraction of Stilbenes and Their Chromatographic Analysis

**Figure 2:**
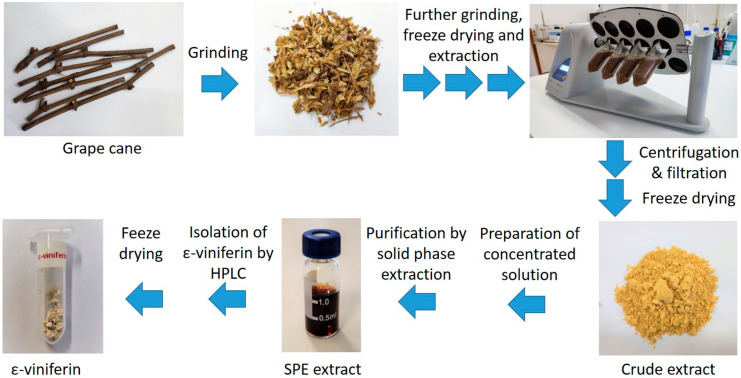
Key steps of grapevine cane processing to obtain crude extract and pure ε-viniferin.

We have developed a simple and efficient method for extracting the stilbenes and isolating ε-viniferin from the canes. In preliminary experiments, we studied the extraction conditions by using single-parameter optimization. Acetonitrile-water ratio and duration of the extraction were considered to estimate the highest yield of ε-viniferin. The whole process extended with the purification and isolation steps are summarized in Figure 2.

**Figure 3:**
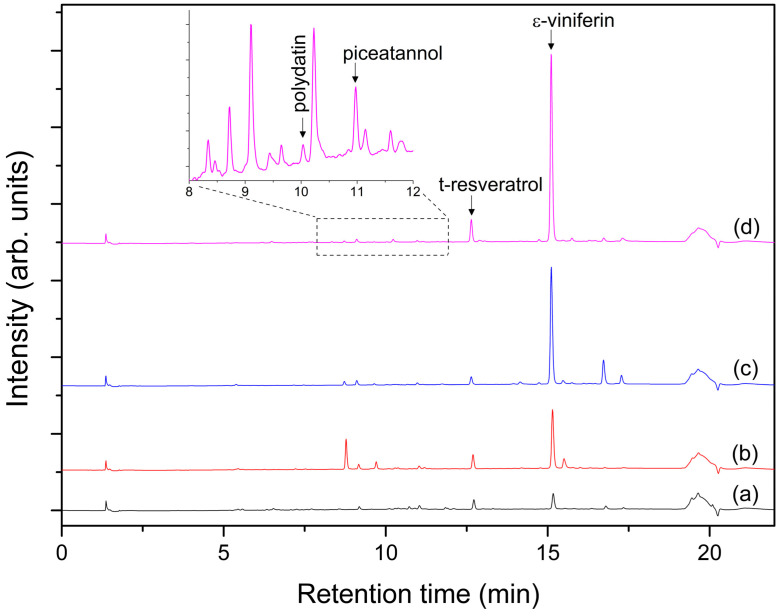
Typical HPLC-DAD chromatograms of cane extracts for (**a**) *Vitis davidii*, (**b**) *Vitis romanetii*, (**c**) *Vitis amurensis*, and (**d**) *Vitis pentagona* species recorded at 306 nm. Inset shows the peaks belonging to polydatin (t_r_ = 10.06) and piceatannol (t_r_ = 11.03 min) on the chromatogram between 8 and 12 min at higher magnification.

Based on preliminary results, a mixture of acetonitrile and water 70:30 *v*/*v*% was the most efficient solvent for extracting stilbenes. Compared to ethanol, acetonitrile has the advantage of a much higher melting point (−45 °C). Therefore, the extract can be directly freeze-dried without the need for an additional evaporation step. Figure 3 compares the chromatograms recorded at 306 nm of cane extracts from different grape species. The stilbene profile is rather simple, and the major stilbene compound for all species was ε-viniferin, while polydatin, piceatannol, and resveratrol were identified at a lower level. Pterostilbene was not detectable in any of the extracts. The chromatograms clearly indicate that the stilbene profile varies depending on the genotype. For instance, the intensity of the peak corresponding to ε-viniferin differs significantly between species.

**Figure 4:**
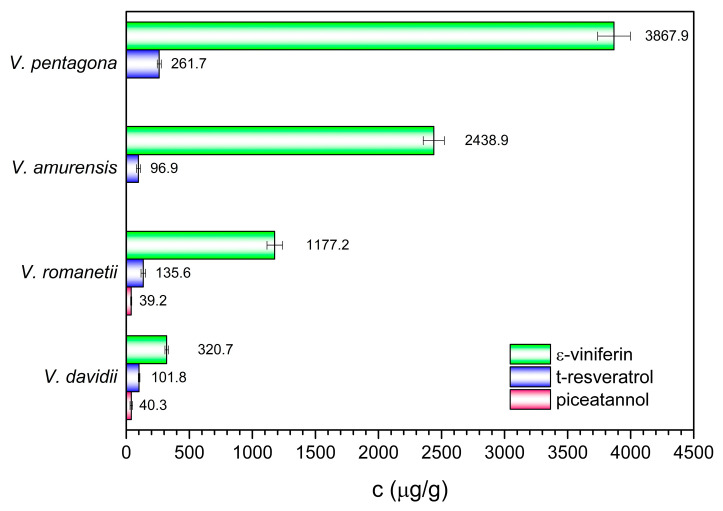
The level of the main stilbenes in grapevine canes for different wild species.

The results of the quantitative analysis are shown in Figure 4. The ε-viniferin content varied within a relatively wide range from ≈ 320 to 3870 µg/g dry weight of cane, and increased in the following order: *V. davidii*, *V. romanetii*, *V. amurensis*, and *V. pentagona*. The level of resveratrol in *V. pentagona* cane was remarkably high compared to other species (261.7 µg/g). Polydatin level was below the limit of quantification (12 µg/g calculated in cane) in all cases. Also, piceatannol was detectable in trace amount for *V. amurensis* and *V. pentagona*, while *V. romanetii* and *V. davidii* contained ≈ 40 µg/g DW.

Stilbenes are phenolic compounds that are produced via the phenylpropanoid and acetate–malonate pathway in a number of plant families that express the stilbene synthase enzyme, including the *Vitaceae* family [48]. They are characterized by a 1,2-diphenylethylene skeleton [49], with resveratrol (3,5,4′-trihydroxy-*trans*-stilbene) representing the leading compound of the group, which can be further biosynthesized to glucosidic derivatives (polydatin, also known as piceid), hydroxyl derivatives (piceatannol), methylated derivatives (pterostilbene), and oligomers (α-viniferin and ε-viniferin), among others [31]. These compounds display several bioactivities, including anticancer [50], antidiabetic [51], anti-inflammatory [52], and anti-angiogenic [53] properties.

During grape production, a considerable amount of cane is remained after pruning, which is either used as compost or burned [27]. However, considering that current emission regulations are in favor of disallowing cane burning [54], several studies pointed out the importance of utilizing these by-products by extracting bioactive compounds, such as stilbenes, which can promote the food, pharmaceutical, and cosmetics industries [35,37,38]. In fact, based on the global production of grapes in 2008, Rayne et al. estimated that extraction of just two stilbenes, namely *trans*-resveratrol and *trans*-ε-viniferin, may reach an economic value of more than USD 30 billion [55].

Our results revealed that main stilbenoids with measurable amounts were ε-viniferin, *trans*-resveratrol, and piceatannol. These results were partially in agreement with Kiselev et al. [56], who measured the stilbene content in different parts of *V. amurensis*, namely the leaves, petioles, berry skins, and seeds, collected in summer and autumn time. In their study, six main compounds were detected, namely *cis*-piceid, *t*-piceid, *t*-ε-viniferin, cis-ε-viniferin, *t*-resveratrol, and *t*-δ-viniferin, where autumn leaves (0.390 mg/g DW) and berry skins (0.249 mg/g DW) scored the highest values of total stilbene content. However, the predominant compound was *t*-piceid in the autumn leaves (0.257 mg/g DW), while in our study it was ε-viniferin (3.9 and 2.4 mg/g in *V. pentagona* and *V. amurensis*, respectively). These results reveal that the stilbene profile of the different parts of the plant differs, furthermore, the ε-viniferin content of the canes is much higher than that of the leaves. Additionally, the stilbene concentrations differ depending on genotype. While ε-viniferin concentration scored 0.32 g/kg DW in *V. davidii* in our study, a previous work reported the *trans*-ε-viniferin concentration in fresh weight as lower than 0.2 g/kg cane FW in *V. davidii* 49 k, while in another genotype (*V. davidii* 2485) it scored 0.6 g/kg cane FW [57]. Moreover, the content of these compounds may vary between cultivars, as shown by Zhang et al., who estimated the concentration of *trans*-resveratrol in canes from seven Chinese regions. The content of *trans*-resveratrol in *V. pentagona* ranged from 700.6 ± 64.9 mg/kg FW for (Lantian, Wangshunshan, Dafeng, Yunxi) that belong to different cultivars and genotypes, to 838 ± 30.9 mg/kg FW in Douan cultivar. On the other hand, Gaoshan, Shuijing Brier, Xuefengshan, Baiyu, Chongyi, Junzi, and Tangwei, which belong to different genotypes and cultivars of *V. davidii*, scored 1048.9 ± 137.9 mg/kg, while in *V. amurensis* varieties (Shuanghong, Shuangyou, Tonghua, and Zuoshan), and the hybrids between *V. amurensis* and *V. vinifera* (Beichun, Beihong, and Gongniang), the authors stated a *trans*-resveratrol estimation of 889.7 ± 62.2 mg/kg FW [58]. These figures, although reported based on the fresh weight, exceeded those recorded in our research (Table S1), as the content of *trans*-resveratrol peaked in *V. pentagona* (261.7 ± 16.21 mg/kg), followed by *V. davidii* (101.8 ± 6.23 mg/kg) and *V. amurensis* (96.9 ± 15.78 mg/kg), which indicates the fluctuations and the inconsistency of the stilbene’s concentrations among grapevine varieties and genotypes. In addition, environmental conditions are key determinants of stilbene content in *Vitis* species, as revealed by a three-year study of thirteen *Vitis* grapevine stilbene-rich shoots. It was demonstrated that the ideal status for stilbene production involves harsh conditions, such as high temperatures, intense sunlight exposure, and low humidity. On the other hand, elevated humidity, which is suitable for fungal infection, increases dimer stilbenes as a defense mechanism [59].

Consequently, stilbenes’ main role is believed to be acting as phytoalexins, compounds that are produced de novo by plants in response to pathogenic infections or stress conditions; hence, they can be regarded as disease resistance markers [60]. A study explored the molecular mechanism that regulates the stilbene biosynthesis in response to infections through inoculating *V. davidii* with grapevine powdery mildew. The findings demonstrated a significant upregulation of *VdMYB1*, a grapevine R2R3-type MYB transcription factor which activates STS genes, leading to increased resveratrol content and enhanced fungal resistance [61].

### 2.3. Antimicrobial Activity of Cane Extracts

**Figure 5:**
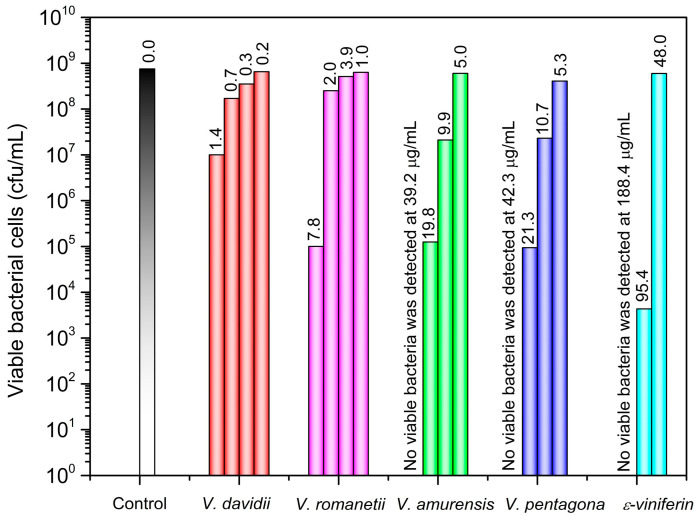
Antilisterial activity of pure ε-viniferin and various grapevine cane extracts after 24 h exposure. The numbers above the columns indicate the ε-viniferin concentrations expressed in µg/mL.

The efficacy of stilbene-rich crude cane extracts and ε-viniferin, the predominant stilbenic compound in the examined grape canes, was tested against a widespread foodborne pathogen, *LM*. In a pilot study, the efficacy of the crude extracts was tested, revealing that 2.4 mg/mL was required to demonstrate antibacterial efficacy. Even with the relatively high concentration, only crude extracts of *V. pentagona* and *V. amurensis* showed bactericidal effects. For antibacterial investigations, the cane extraction procedure was scaled up with the modification of experimental conditions (see Section 3.5). *V. pentagona* was further purified using solid-phase extraction and preparative HPLC to yield high-purity ε-viniferin, the main stilbene that is presumed to be the active ingredient (see Section 3.6). Figure 5 demonstrates the number of living bacterial cells after 24 h exposure to ε-viniferin-rich crude extracts and pure ε-viniferin. Both *V. amurensis* and *V. pentagona* extracts showed bactericidal effects at 39.2 µg/mL and 42.3 µg/mL of ε-viniferin, respectively. On the other hand, isolated pure ε-viniferin (purity > 95%) was not effective at this level, and it required reaching 188.4 µg/mL to kill the bacteria. Neither *V. davidii* nor *V. romanetii* extracts was able to eliminate *LM* completely, with the latter being partially more effective. The content of ε-viniferin in these species is lower, yet *V. romanetii* exhibited antibacterial activity at 7.8 µg/mL, comparable to the efficacy of *V. amurensis* at 19.8 µg/mL and *V. pentagona* at 21.3 µg/mL of ε-viniferin. This finding reflects the different stilbene profiles of the extracts and suggests the presence of other compounds in *V. romanetii* that possess antibacterial activity, such as piceatannol, with its listerial growth inhibition percentage reported as (89.1 ± 0.8%) at 0.5 mM concentration [62].

Our results show that there is a positive correlation between ε-viniferin content of the extract and the antilistirial efficacy. To the best of our knowledge, no former study examined the cane extract activity against *LM*. However, one study tested the antibacterial efficacy of ethyl acetate extracts of Spanish *V. vinifera* grape shoots against closely related nonpathogenic bacteria, *Listeria innocua*, in addition to other Gram-positive and Gram-negative bacteria [63]. The extract showed efficacy against tested microorganisms, with minimum inhibitory concentrations (MIC) and minimum bactericidal concentrations (MBC) values for *Listeria innocua* of (5 mg/mL) and (10 mg/mL), respectively, with no conclusion about a specific antibacterial factor due to the complexity of the extracts.

**Figure 6:**
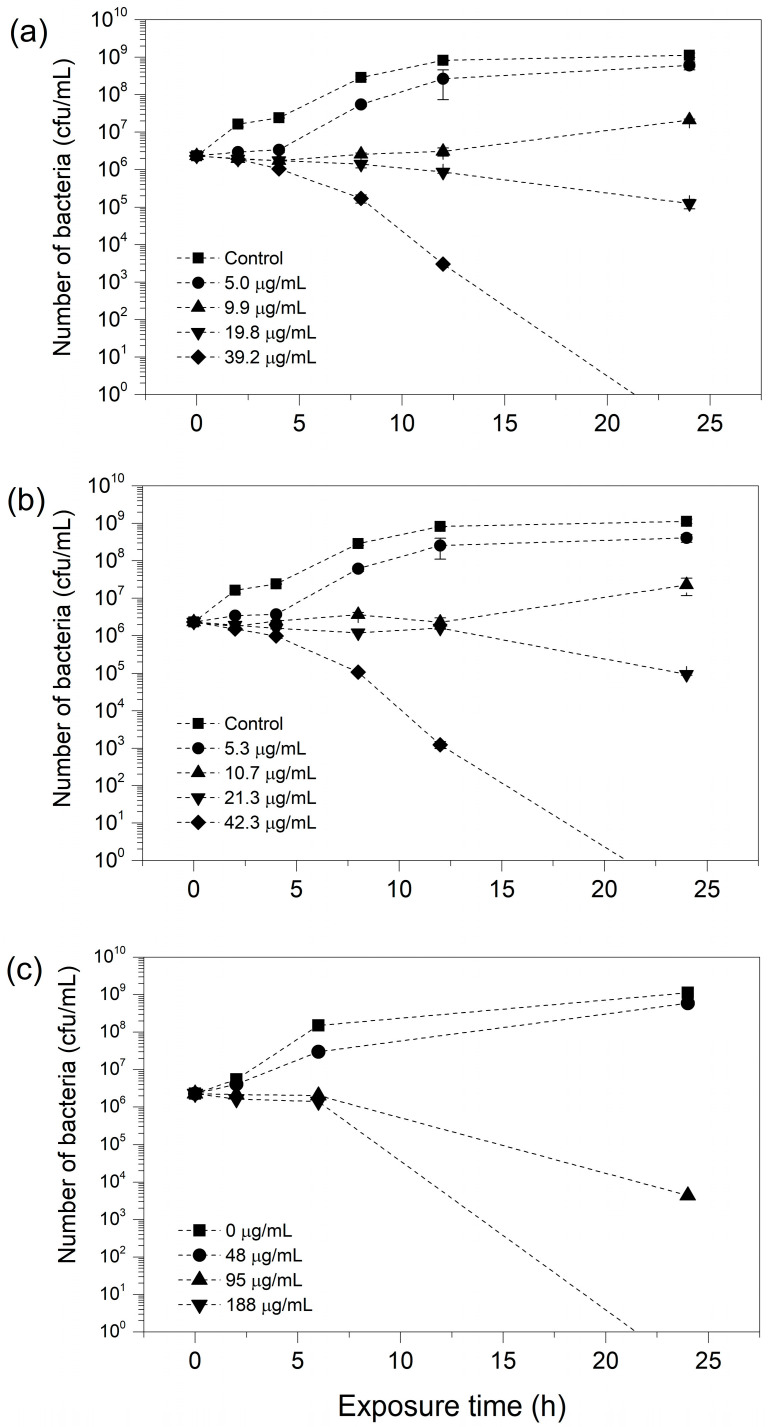
Time–kill curves of *Listeria monocytogenes* exposed to (**a**) *V. amurensis*, (**b**) *V. pentagona* cane extracts containing ε-viniferin at different concentrations, and (**c**) pure ε-viniferin.

*V. amurensis* and *V. pentagona* crude extracts, as well as ε-viniferin, completely eliminated the bacteria after 24 h exposure. Based on the HPLC analysis, these bacteria suspensions contained ε-viniferin at 39.2 µg/mL, 42.3 µg/mL, and 188.4 µg/mL, respectively. Following these results, we conducted a time–kill assay to estimate the time needed for the bactericidal effect. The number of bacteria started to drop at 2 h, as shown in Figure 6, and continued to decrease in the subsequent hours, as detailed in Tables S2 and S3. After 24 h, *V. amurensis* and *V. pentagona* extracts were able to eradicate the bacteria in their highest ε-viniferin concentration, while pure ε-viniferin required almost fivefold increased concentration to kill the bacteria, suggesting that other compounds in the cane extract possess antibacterial activity. For example, resveratrol at 200 µg/mL showed an inhibitory effect against *LM* after exposures lasting 24 h [64], and the presence of both resveratrol and ε-viniferin in the cane extract might explain the synergetic effect, and the lower ε-viniferin concentration needed to kill *LM*. It is worth noting that while resveratrol was only bacteriostatic according to the literature, we demonstrated that ε-viniferin at the same concentration was bactericidal, i.e., having stronger antibiotic activity.

While the number of studies examining the antibacterial efficacy of ε-viniferin is still limited [38,44,45,46], extensive research has been conducted on the effectiveness of other stilbenes against several pathogens. For instance, resveratrol inhibited *Escherichia coli* growth through binding to ATP synthase and suppression of oxidative phosphorylation [65]. Additionally, this compound targeted the biofilm formation of several Gram-positive and Gram-negative bacteria [66]. Several stilbenes have also been tested against *Staphylococcus aureus* (*S. aureus*), where pterostilbene showed the lowest minimum inhibitory concentration (MIC) at (32–64 µg/mL), followed by piceatannol (64–256 µg/mL) and pinostilbene (128 µg/mL). Other compounds including 3′-hydroxypterostilbene, isorhapontigenin, and rhapontigenin ranged from 128 to 256 µg/mL, while resveratrol required more than 512 µg/mL [67]. Piceatannol exhibited MIC of 128 µg/mL and MBC of 256 µg/mL against *S. aureus* in a study conducted in 2022 [68], and its synergetic effect with ciprofloxacin has been illustrated in the same study, which indicated that piceatannol affected the proton motive force of *S. aureus*, leading to escalated bacterial sensitivity to ciprofloxacin.

To conclude, we highlight the importance of grapevine canes as a valuable source to be utilized as a natural food preservative, particularly in ready-to-eat meals, that hold high risk for foodborne pathogens such as *LM*. We also provide essential information for clinical research and pharmaceutical manufacturers to use ε-viniferin as an effective antibiotic against *LM*. We also draw attention to the need for further investigation to compare the antibacterial activity of ε-viniferin and other stilbenes, solely and together, to confirm the additive efficacy that was reported in this paper.

## 3. Materials and Methods

### 3.1. Chemicals and Reagents

Acetonitrile and methanol (Promochem Optigrade, LGC Standards GmbH, Wesel, Germany) were ≥ 99.9%, gradient grade, suitable for HPLC. Polydatin (≥99.0%), piceatannol (≥98.0%), *trans*-resveratrol (≥99.0%), ε-viniferin (≥98.0%), and pterostilbene (≥95.0%) were purchased from Extrasynthese (Genay, France). Acetic acid (≥99.9%) was purchased from Molar Chemicals Ltd., Halásztelek, Hungary. High-purity deionized water was obtained using a LaboStar 7 TWF-UV ultrapure water system (SG Wasseraufbereitung und Regenerierstation GmbH, Barsbüttel, Germany).

### 3.2. Experimental Site and Plant Material

One-year-old fully matured, woody shoots, hereinafter canes were collected from thirteen-year-old vines of *Vitis davidii*, *Vitis romanetii*, *Vitis amurensis*, and *Vitis pentagona*. All the grapevine trunks were grown under non-irrigated open-field conditions on the south-facing slopes of Mecsek Hills, Hungary (latitude: 46°04′ N, longitude: 18°11′ E, 150 m a.s.l.), at the central station of the Research Institute of Viticulture and Oenology, University of Pécs, Hungary. Vines were grafted on commonly used rootstock varieties ‘T5C’ (*Vitis berlandieri* × *Vitis riparia*) and were planted with 2 × 1 m vine spacing with a North–South row direction in a mid–high cordon trellis system. For all the aforementioned *Vitis* species, five individual vines were chosen, and three canes with 6–8 nodes were collected from each vine.

### 3.3. Genetic Fingerprinting and Ampelographic Assessment of Grapevine Species

DNA was extracted from canes with the Plant DNA Mini Kit (Peqlab, Erlangen, Germany) following the supplier’s instructions. Samples were genotyped with the nine GrapeGen06 markers VVS2, VVMD5, VVMD7, VVMD25, VVMD27, VVMD28, VVMD32, VrZAG62, and VrZAG79, listed in the *Vitis* International Variety Catalogue [69], to distinguish and identify grapevine cultivars as well as individuals of wild species. PCR amplification, fragment length determination, and allele size adaption were conducted according to Perko et al. [70].

Furthermore, morphology of all individuals of the *Vitis* spp. was checked in the field regarding species-typical ampelographic traits.

### 3.4. Preparation of Cane Extracts for HPLC Analysis

The collected canes were cut into small pieces using an electric garden shredder (FZD 4020-E). To obtain a smaller grain size, the resulting grist was further ground with a simple coffee grinder, and then freeze-dried for 24 h using a ScanVac CoolSafe 110-4 Freeze Dryer (LaboGene ApS, Allerod, Denmark). In total, 1 mL of a mixture of acetonitrile-water (70/30 *v*/*v*%) was added to 50 mg of the freeze-dried powder sample of each species and then sonicated for 15 min using an Elma Transsonic T 460 ultrasonic bath with a noise frequency of 35 kHz (Singen/Hohentwiel, Germany). The resulting suspensions were centrifuged at 20,660× *g* for 10 min, and subsequently the extraction of the sediments was repeated two times. The obtained supernatants were merged in a 5.0 mL volumetric flask. For each species, three extracts were prepared. Before HPLC analysis, the solutions were filtered through a 0.22 µm PES syringe filter (FilterBio^®^, Labex Ltd., Budapest, Hungary).

### 3.5. Preparation of Crude Extract for Antibacterial Tests

To obtain a larger amount of ε-viniferin, the following extraction procedure was carried out with the required number of repetitions, using woody cane samples. In total, 40 mL of 70:30 *v*/*v*% acetonitrile and water mixture was added to 2.5 g of fine powder of ground freeze-dried cane, and then was sonicated for 30 min. The extraction was continued for 24 h using a PTR-60 vertical multifunction rotator (Grant Instruments Ltd., Cambridgeshire, UK). Finally, the obtained suspension was centrifuged at 21,380× *g* for 10 min and filtered through using a 0.22 µm PES syringe filter (FilterBio^®^, Labex Ltd., Budapest, Hungary). The filtrate was freeze-dried for 24 h, and the resulting material was designated as a crude extract. Prior to the antibacterial tests, all of the crude extracts were analyzed using HPLC.

### 3.6. Purification of Crude Extract and Isolation of ε-Viniferin

The crude extract of *V. pentagona* was purified with solid-phase extraction using Strata PAH 1.5 g/6 mL tubes. In total, 100 mg of crude extract powder was dissolved in 500 µL of 5% methanol. The purification steps were as follows: conditioning, 3 mL of methanol and then 3 mL of water; sample injection, 500 µL of sample; washing, 3 mL of water and then 3 mL of 50% methanol; elution, 2 × 3 mL of a mixture of water and acetonitrile (70:30 *v*/*v*). Eluate was freeze-dried for 24 h, and the obtained powder was designated as SPE-purified cane extract. High-purity ε-viniferin was isolated from SPE-purified cane extract with preparative chromatography using a Kinetex^®^ 5 µm XB-C18 100 Å, 250 × 10.0 mm LC column (Phenomenex, Torrance, CA, USA) semi-preparative LC column. For the preparative chromatography, Shimadzu Prominence UFLC system (Shimadzu Co., Kyoto, Japan) consisting of an online degassing unit (DGU-20A5R), pump (LC-20AD), column oven (CTO-20AC), autosampler (SIL-20AC HC), diode array detector (SPD-M20A), and fraction collector (FRC-10A) was used. The column temperature was kept at 25 °C. Gradient elution was applied using 0.5% (*v*/*v*) acetic acid (A) and a mixture of acetonitrile (99.5% *v*/*v*) and acetic acid (0.5% *v*/*v*) (B). Gradient elution started at 100% (*v*/*v*) A, ramping up to 50% B over 20 min. The column was washed with 100% (*v*/*v*) B for 1 min, and then equilibrated with 100% (*v*/*v*) A for 5 min. The flow rate of the mobile phase was kept at 5.0 mL/min; the volume of the injected SPE-purified cane extract (200 mg/mL) was 100 μL.

### 3.7. Chromatographic Analysis of Stilbenes

Chromatographic analysis was performed on the aforementioned HPLC system, while the separation was conducted on a Kinetex^®^ 2.6 µm XB-C18 100 Å, 100 × 4.6 mm LC column (Phenomenex, Torrance, CA, USA). The column temperature was kept at 25 °C. Gradient elution was applied using 0.5% (*v*/*v*) acetic acid (A) and a mixture of acetonitrile (99.5% *v*/*v*) and acetic acid (0.5% *v*/*v*) (B). Gradient elution started at 100% (*v*/*v*) A, ramping up to 56% B over 20 min. The flow rate of the mobile phase was kept at 1.0 mL/min; the volume of the injected sample was 5 μL. For the quantitative evaluation, the absorbance values acquired at 306 nm were used. Calibration curves were obtained by measuring polydatin, piceatannol, *trans*-resveratrol, ε-viniferin, and pterostilbene with known concentrations (Table S4). Spiking of the extracts with reference materials for all matrices was performed to check the compound identity. The results were expressed in µg stilbene per mg dry weight (DW) of cane or its solid extract.

### 3.8. Antimicrobial Experiments

#### 3.8.1. Bacterial Strain and Culture Conditions

*Listeria monocytogenes* OEK ATCC35152 suspension was prepared from a single colony of *LM* (previously grown on blood-based (BB) agar) in Luria–Bertani broth (LB) to achieve an optical density (≈0.2) compared to the control (LB). Phosphate-buffered saline (PBS) was used to dilute the incubated bacterium suspensions prior to counting the bacterium colonies on BB agar plates. During the experiment, all tested tubes were incubated in a shaking incubator (aerobic conditions, 37 °C). Agar plates were incubated aerobically at 37 °C.

#### 3.8.2. Antibacterial Test and Time–Kill Assay

In total, 100 mg of crude extract from each species was separately dissolved in 1.0 mL of 50% *v/v* ethanol. The obtained solutions were analyzed using HPLC-DAD measurements to check their stilbene profile prior to the antibacterial experiments. Then, a series of dilutions were prepared by adding 50, 25, 12.5, or 6.25 µL of each extract into a sterile tube containing 2 mL of LB medium and 20 µL of the bacterial suspension. The final ethanol concentrations were 1.2%, 0.6%, 0.3%, and 0.15% *v*/*v*, respectively. The same procedure was conducted for pure ε-viniferin (purity ≥ 95%), starting from a stock solution of 7.8 mg/mL prepared in 50% ethanol. A series of dilutions were prepared from the stock solution, with final concentrations of 188.4, 95.3, and 48.0 µg/mL of ε-viniferin, and 1.2%, 0.6%, and 0.3% of ethanol, respectively. In parallel, a control sample was tested, adding 50 µL of 50% ethanol instead of the extract, and reaching a final concentration of 1.2% *v/v* of ethanol. The number of living cells in the initial bacterial stock suspension was 3.3 × 10^8^ CFU/mL (details are described in Section 3.8.1), while it scored ~2.3 × 10^6^ CFU/mL in the tested suspensions. After incubating the suspension for 24 h, serial dilutions in PBS were prepared from each tube, and 10 µL of each dilution was dropped and run off onto (BB) agar plates to count the colonies.

Time–kill curve experiments were performed for 24 h using only the bactericidal agents, i.e., *V. amurensis* and *V. pentagona* cane extracts and pure ε-viniferin. The aforementioned steps and concentrations were followed, and living cells were counted in the suspensions after incubation with *V. amurensis* and *V. pentagona* extracts for 0, 2, 4, 8, 12, and 24 h, whereas for ε-viniferin, the living cells were counted after 0, 2, 6, and 24 h of exposure.

### 3.9. Statistical Analysis

Statistical analysis was performed using SPSS 26.0 for Windows (SPSS Inc., Chicago, IL, USA). After checking the normal distribution and homogeneity of variances, One-Way ANOVA (Analysis of Variance) was applied at the level of significance *p* < 0.05. Pairwise comparisons were conducted using Tukey’s HSD post hoc test. Data are expressed as the mean of three measurements ± standard deviation.

## 4. Conclusions

The stilbene profile of wild grape canes is highly genotype-dependent. For the species studied, the predominant stilbene in the canes was ε-viniferin. Resveratrol was found in lower concentrations, while polydatin and piceatannol were detected in trace amounts. No pterostilbene was detected. The cane extracts were found to be effective against *Listeria monocytogenes* in vitro depending on their ε-viniferin level. The higher the ε-viniferin content, the greater the antimicrobial effect observed. Taking into account the ε-viniferin concentrations, the efficacy of crude extract was superior to that of pure ε-viniferin. This indicates that other antibacterial compounds (e.g., resveratrol and piceatannol) besides ε-viniferin were also present in the extracts. As a consequence of the extract’s complexity, it may contain components that promote or potentiate the antilisterial effect of ε-viniferin. Further studies are necessary to confirm a possible synergism between the individual compounds. *V. amurensis* and *V. pentagona* canes are excellent natural sources of ε-viniferin, and their extracts may have promising applications in both food and pharmaceutical industries.

## Figures and Tables

**Table 1:** Analyzed *Vitis* species with genotypic data of nine nSSR markers.

*Vitis* Species/Reference Variety	VVMD27:1	VVMD27:2	VVS2:1	VVS2:2	VVMD7:1	VVMD7:2	VVMD5:1	VVMD5:2	VRZAG62:1	VRZAG62:2	VRZAG79:1	VRZAG79:2	VVMD28:1	VVMD28:2	VVMD32:1	VVMD32:2	VVMD25:1	VVMD25:2
*V. pentagona*	186	195	129	171	237	238	236	250	174	196	253	261	238	242	242	242	249	249
*V. romanetii*	184	186	129	129	243	245	248	250	214	214	247	249	220	238	248	248	251	255
*V. amurensis*	188	190	139	141	241	245	236	240	186	188	245	257	248	252	238	248	237	237
*V. davidii*	178	192	133	141	237	239	242	244	184	188	241	241	226	258	232	242	237	247

## Data Availability

The unpublished data which may provide additional information in order to understand the present research are accessible from the corresponding author.

## Supplementary Materials

The following supporting information can be downloaded at: https://www.mdpi.com/article/10.3390/molecules29153518/s1, Table S1: Stilbene content of canes for different *Vitis* species; Table S2: The impact of the *V. amurensis* and *V. pentagona* cane extracts on the viable bacterial cells after exposure lasting between 0 and 24 h; Table S3: The effect of pure ε-viniferin on the viable bacterial cells after exposure lasting between 0 and 24 h; Table S4: Equations of calibration curves of individual stilbenes.
