# Supplementary material for: Wild Vitis Species as Stilbenes Sources: Cane Extracts and Their Antibacterial Activity against Listeria monocytogenes
Source: Molecules. 2024 Jul 26;29(15):3518. doi: 10.3390/molecules29153518 (PMC11314568; doi:10.3390/molecules29153518)
Supplement: Supplementary file 1 [file molecules-29-03518-s001.zip › molecules-3098010-supplementary.pdf]

Wild *Vitis* Species as Stilbenes Sources: Cane Extracts and Their Antibacterial Activity against *Listeria monocytogenes*

Okba Hatem [1,2], Anita Steinbach [3], György Schneider [3], Franco Röckel [4] and László Kőrösi [5,*]

[1] Doctoral School of Health Sciences, Faculty of Health Sciences, University of Pécs, H-7622 Pécs, Hungary
[2] Department of Biochemistry and Medical Chemistry, Medical School, University of Pécs, H-7624 Pécs, Hungary
[3] Department of Medical Microbiology and Immunology, Medical School, University of Pécs, Szigeti Street 12, H-7624 Pécs, Hungary
[4] Julius Kühn Institute (JKI), Institute for Grapevine Breeding Geilweilerhof, 76833 Siebeldingen, Germany
[5] Research Institute for Viticulture and Oenology, University of Pécs, H-7634 Pécs, Hungary
* Correspondence: korosi.laszlo@pte.hu

Supplementary material

Table S1: Stilbene content of canes for different *Vitis* species. Data are shown as mean of three measurements ± SD. Similar superscript letters within the same column indicate significant differences between grapevine varieties (p <0.05).

	Stilbene content (µg/g)			
Vitis species	piceid	piceatannol	resveratrol	ε-viniferin
V. davidii	1.0 ± 0.1 ^{abc}	40.3 ± 10.1 ^{ab}	101.8 ± 6.2 ^{a}	320.7 ± 14.0 ^{abc}
V. romanetii	3.0 ± 0.1 ^{ade}	39.2 ± 4.2 ^{cd}	135.6 ± 18.7 ^{b}	1177.2 ± 61.5 ^{ade}
V. amurensis	8.4 ± 0.3 ^{bdf}	7.0 ± 0.1 ^{ac}	96.9 ± 15.8 ^{c}	2438.9 ± 83.9 ^{bdf}
V. pentagona	6.7 ± 0.5 ^{cef}	7.5 ± 0.1 ^{bd}	261.7 ± 16.2 ^{abc}	3867.9 ± 129.7 ^{cef}

Table S2: The impact of the *V. amurensis* and *V. pentagona* cane extracts on the viable bacterial cells after exposure lasting between 0 and 24h. Data are shown as mean of three measurements ± SD. Similar superscript small letters indicate pairwise significance within a raw (over time points), while similar superscript capital letters indicate pairwise significance within a column (at the same time point) (p <0.05).

concentration of ε-viniferin in *Vitis* crude extracts (µg/mL)		CFU/ml × 10^6					
		**0h**	**2h**	**4h**	**8h**	**12h**	**24h**
V. amurensis	39.2	2.35 ± 0.10 ^{abcde}	1.90 ± 0.10 ^{afghiA}	1.05 ± 0.05 ^{bfijkABCD}	0.17 ± 0.01 ^{cgilmABC}	0.03 ± 0.00 ^{dhjlABC}	0 ± 0 ^{eikmABC}
	19.8	2.35 ± 0.10 ^{abcde}	1.90 ± 0.10 ^{afghB}	1.70 ± 0.05 ^{bijkEFG}	1.40 ± 0.02 ^{cfilmnDEF}	0.85 ± 0.00 ^{dgjlDEF}	0.13 ± 0.00 ^{ehkmnDEF}
	9.9	2.35 ± 0.10 ^{a}	1.95 ± 0.15 ^{bC}	1.75 ± 0.05 ^{cdHIJ}	2.55 ± 0.15 ^{eGHI}	3.05 ± 0.05 ^{cfGHI}	21.00 ± 0.10 ^{abdefGHI}
	5.0	2.35 ± 0.10 ^{ab}	2.95 ± 0.05 ^{cdD}	3.35 ± 0.25 ^{efAEHKL}	55.00 ± 2.50 ^{ghADGJKLM}	265.00 ± 13.50 ^{acegiADGJKLMN}	600.00 ± 10.00 ^{bdfhiADGJKLMN}
V. pentagona	42.3	2.35 ± 0.10 ^{abcde}	1.53 ± 0.03 ^{afghiE}	0.99 ± 0.08 ^{bfjklKMNOPQ}	0.11 ± 0.01 ^{cgjmnJNO}	0.01 ± 0.00 ^{dhkmKOP}	0 ± 0 ^{eilnJOP}
	21.3	2.35 ± 0.10 ^{abcde}	1.91 ± 0.01 ^{afghiF}	1.57 ± 0.06 ^{bfjkLNRS}	1.20 ± 0.01 ^{cgjlmnKPQ}	1.6 ± 0.01 ^{dhlLQR}	0.09 ± 0.00 ^{eikmnKQR}
	10.7	2.35 ± 0.10 ^{a}	1.77 ± 0.03 ^{bG}	2.48 ± 0.10 ^{cBOTU}	3.63 ± 0.15 ^{dLRS}	2.28 ± 0.07 ^{eMS}	23.00 ± 1.13 ^{abcdeLS}
	5.3	2.35 ± 0.10 ^{ab}	3.43 ± 0.09 ^{cdH}	3.70 ± 0.26 ^{efCFIPRTV}	62.00 ± 5.50 ^{gBEHNPR}	256.67 ± 14.50 ^{aceBEHOQST}	406.60 ± 9.65 ^{bdfgBEHMOQST}
**Control**	0	2.35 ± 0.10 ^{abc}	16.33 ± 0.25 ^{defABCDEFGH}	24.27 ± 1.10 ^{ghiDGJMQSUV}	286.67 ± 25.54 ^{adgjkCFIMOQS}	820.00 ± 26.46 ^{behjlCFINPRT}	1133.33 ± 99.75 ^{cfiklCFNPRT}

Table S3: The effect of pure ε-viniferin on the viable bacterial cells after exposure lasting between 0 and 24h. Data are shown as mean of three measurements ± SD. Similar superscript small letters indicate pairwise significance within a raw (over time points), while similar superscript capital letters indicate pairwise significance within a column (at the same time point) (p <0.05).

**pure ε-viniferin** (μg/mL)	**CFU/ml × 10^6**			
	**0h**	**2h**	**6h**	**24h**
188	2.30 ± 0.10^{abc}	1.63 ± 0.02^{adAB}	1.40 ± 0.01^{beA}	0 ± 0^{cdeAB}
95	2.30 ± 0.10^{a}	2.17 ± 0.15^{bCD}	2.03 ± 0.15^{cB}	0.01 ± 0.00^{abcCD}
48	2.30 ± 0.10^{ab}	4.10 ± 0.36^{cdACE}	30.00 ± 0.50^{aceC}	$596.67 \pm 15.27^{bdeACE}$
0	2.30 ± 0.10^{a}	5.53 ± 0.42^{bBDE}	150.00 ± 13.58^{cABC}	$1133.33 \pm 112.28^{abcBDE}$

Table S4: Concentration ranges of individual stilbenes for the calibration, equations of calibration curves, and correlation coefficients. Five concentration levels were used for the calibration of each analyte.

Compound	Range of calibration μg/mL	Equation	R^2
piceid	1-5	y = 18088.1x – 954.57	0.9999407
piceatannol	1-5	y = 20635.6x – 2149.53	0.9996082
trans-resveratrol	1-5	y = 27274.5x – 2223.27	0.9999355
ε-viniferin	1-100	y = 15312.3x – 2098.50	0.9999309
pterostilbene	1-5	y = 29695.9x – 1196.42	0.9999705
